# 

**Published:** 1887-03

**Authors:** 


					﻿MARCH, 1887.
OLD SERIES
VoLXXV
NEW SERIES
Vol. IV.-No. I.
lass <>'


4nUBNA^

W.F.WESTMOR£i::%ND.Hj
H.V.MMlLLER.M,D.LL.D.J|r
JAMES AGRAY. MJJ.'l
ri^BUSMEOBy «IAMESRHARRISQN&eJ
________________SSTLAMTAaCA.
SUBSCRIPTION: 12.50 A YEAR. '
				

